# Is idiopathic toe walking a symptom of sensory processing dysfunction?

**DOI:** 10.1186/1757-1146-4-S1-P59

**Published:** 2011-05-20

**Authors:** Cylie Williams, Paul Tinley, Michael Curtin

**Affiliations:** 1 Charles Sturt University, Albury, NSW, 2640, Australia; 2 Southern Health - Cardinia Casey Community Health, Cranbourne, VIC, 3977, Australia; 3 Peninsula Health – Community Health, Frankston, 3199, Australia

## Background

It is understood that toe walking involves the absence or limitation of heel strike in the contact phase of the gait cycle. When there is no medical cause of the gait pattern, a diagnosis of idiopathic toe walking (ITW) is made. Although there has been limited research into the pathophysiology of ITW, there has been an increasing number of references proposing that this gait pattern may be linked to sensory processing dysfunction (SPD). The purpose of this study was to investigate and profile 30 children with an ITW gait compared to 30 peers with a typical gait, using both qualitative and quantitative data.

## Methods

Participants were assessed using the: (i) Bruininks-Oseretsky Test of Motor Proficiency (BOT-2), (ii) The Sensory Profile, (iii) Sensory Integration and Praxis test (SIPT), (iv) Vibration Perception Threshold (VPT) and (v) interview with parents about social factors of toe walking

## Results

Results of testing 33 children thus far have determined children with an ITW gait have lower vibration perception thresholds, a preponderance to be left handedness, and notable differences in sensory seeking and modulation scores as measured by the Sensory Profile. The BOT-2 scores found children who toe walked scored overall lower in the composite scores for fine motor and balance tasks; however, they generally scored above average in the strength sub-test. SIPT results found a significant different in post-rotary nystagmus. Parents discussed excitement and anxiousness as being factors that triggered ITW.

## Conclusions

These early results support the theory that the children who had an ITW gait differ in vestibular and tactile processing from the children who had a typical gait. The identification of these differences may assist allied health practitioners understand the gait pattern development and to plan more effective treatment methods.

